# Alterations in the protein lactylation landscape of sperm from patients with varicocele-associated asthenozoospermia

**DOI:** 10.3389/fendo.2026.1791920

**Published:** 2026-06-23

**Authors:** Heran Cao, Xiaohua Liu, Hua Nie, Shenghui Zhu, Shujuan Liu, Yu Zhou, Chunjie Ma, Huang Liu, Weibing Qin

**Affiliations:** 1 NHC Key Laboratory of Male Reproduction and Genetics, Guangdong Provincial Reproductive Science Institute, Guangzhou, China; 2 Innovation Centre for Advanced Interdisciplinary Medicine, The Fifth Affiliated Hospital of Guangzhou Medical University, Guangzhou, Guangdong, China

**Keywords:** asthenozoospermia, glycolysis, protein lactylation, sperm motility, varicocele

## Abstract

**Backgrounds:**

Varicocele is a leading cause of male infertility, primarily manifesting as asthenozoospermia. The underlying molecular mechanisms remain unclear, and the potential role of protein lactylation in this condition is unknown.

**Methods:**

We performed a comprehensive lactylome analysis using data-independent acquisition (DIA) mass spectrometry on paired sperm samples collected from patients with varicocele-associated asthenozoospermia before and after treatment. Differential lactylation analysis and bioinformatic enrichment analyses were conducted.

**Results:**

Disrupted lactate metabolism was observed in asthenozoospermic sperm, with decreased intracellular L-lactate and elevated seminal plasma L-lactate. This was concomitant with a global reduction in protein lactylation, particularly in the sperm midpiece and tail. Lactylome profiling identified 2,699 lactylation sites on 1,216 proteins. Comparison of pre- and post-treatment samples revealed 133 differentially lactylated proteins, which were significantly enriched in pathways critical for sperm motility, including glycolysis/gluconeogenesis, microtubule-based movement, and flagellar assembly. Notably, lactylation abundance of key proteins (TEKT3, AKAP4, TUBA1A, AKAP3, TUBB4B) were significantly downregulated.

**Conclusions:**

This study presents an initial characterization of the protein lactylation landscape in human sperm, revealing​ its alteration in the context of varicocele-associated asthenozoospermia. The findings suggest that dysregulation of the lactate-lactylation axis correlates with impaired sperm motility, potentially through concurrent alterations in energy metabolism and flagellar structure. This correlation underscores specific lactylation events that warrant future mechanistic investigation.

## Introduction

Infertility represents a significant contemporary reproductive health issue, with male factors contributing to approximately 50% of cases (1, 2). Among various etiologies, varicocele is one of the most common identifiable and treatable causes of male infertility, affecting about 15% of the general population (3). This condition impairs spermatogenesis and reduces sperm quality through mechanisms such as elevated scrotal temperature, localized hypoxia, and oxidative stress (4). Asthenozoospermia, characterized by reduced sperm motility, is a primary clinical manifestation of male infertility and often leads to difficulties in natural conception (5). Evidence indicates that varicocele is the most frequent identifiable cause of asthenozoospermia and is strongly associated with impaired sperm motility (6). Although varicocele is highly prevalent among infertile men, its precise pathophysiological mechanisms remain incompletely understood.

Current evidence indicates that varicocele is associated with a significant decline in adenine nucleotide energy reserves and impaired spermatogenesis in rat models (7). Proteomic analyses have further revealed disruptions in pathways related to energy metabolism, oxidative stress, and mitochondrial function in the semen and sperm of affected patients (8). However, conventional diagnostic approaches such as semen analysis and DNA fragmentation testing are inadequate for detecting these subcellular and molecular alterations, limiting their utility in clarifying pathological mechanisms and predicting treatment outcomes (9). Although varicocelectomy is a commonly employed intervention, its therapeutic efficacy remains inconsistent, partly due to the complex and poorly understood pathophysiology. Critical molecular alterations, including mitochondrial dysfunction, aberrant cell death, and post-translational modifications, often go undetected (10).

In recent years, metabolism-associated post-translational modifications (PTMs) have gained increasing attention for their central role in regulating cellular function (11, 12). Among these, lactylation has emerged as a novel metabolite-driven epigenetic and metabolic modification, directly mediated by lactate (13). It has been shown to profoundly influence cellular processes in cancer, immunology, and neurobiology by modulating metabolic enzyme activity and gene transcription (14–16). Recently, a foundational study by Yan et al. confirmed the existence of protein lactylation in human sperm, identifying 220 lactylated proteins enriched in glycolytic pathways and sperm flagellar structure (17). Crucially, they reported a global downregulation of lysine lactylation in asthenozoospermic sperm and established a functional link between hypo-lactylation of a key glycolytic enzyme (PGK2) and impaired sperm motility (17). However, given that sperm motility is dependent on glycolysis (18, 19), which generates lactate as an end product, the potential presence of lactylation in sperm and its possible role in varicocele-associated asthenozoospermia represent an intriguing and unexplored area of research. It is important to note that varicocele-induced subfertility is a multifactorial syndrome (8). While impaired spermatogenesis leading to reduced sperm counts is a well-documented effect, likely mediated by factors such as elevated scrotal temperature (20) and oxidative stress (21), the post-testicular, functional defects in mature sperm—particularly asthenozoospermia—are equally critical for fertilization success (22). This study specifically focuses on this post-spermatogenic, functional dimension. We posit that understanding the molecular alterations in mature spermatozoa themselves, such as dysregulated PTMs, is essential to fully elucidate the pathophysiology and to identify potential targets for improving sperm function.

Given the variable outcomes of varicocelectomy, we deliberately employed a paired, pre- and post-treatment study design​ using samples from patients who exhibited a clear improvement in semen parameters (particularly motility) following surgery. This approach serves a specific purpose: it allows us to maximize the likelihood of identifying lactylation changes that are functionally linked to the reversible component of sperm dysfunction in varicocele. By focusing on “responders,” we aim to correlate dynamic shifts in the lactylome with concurrent recovery of motility, thereby filtering out background noise and strengthening the association between lactylation and a treatable form of asthenozoospermia. Therefore, this study aimed to delineate the landscape of sperm protein lactylation in patients with varicocele-associated asthenozoospermia to uncover its underlying pathophysiological mechanisms. We performed quantitative lactyl-proteomics using data-independent acquisition (DIA) on paired sperm samples collected from patients with varicocele-associated asthenozoospermia before and after treatment to characterize the protein lactylation landscape. Our study unveiled a coordinated dysregulation of lactate metabolism and protein lactylation in asthenozoospermic sperm. The hypo-lactylation, prominently observed in proteins involved in glycolysis and flagellar integrity, suggests a novel mechanism impairing the sperm’s energy supply and motility apparatus. These findings highlight lactylation as a potential​ regulatory axis in male infertility, providing fresh insights into the molecular pathogenesis of varicocele and identifying specific lactylation targets for future mechanistic investigation.

## Methods

### Patient recruitment and clinical characteristics

This study enrolled male patients (n=10) clinically diagnosed with varicocele (VC) accompanied by asthenozoospermia according to the WHO 5th edition laboratory manual. All patients underwent high ligation varicocelectomy. For in-depth lactylomic profiling, we selected three matched pairs of pre- and post-treatment sperm samples (n=3) from this cohort. The selection criteria were (1): successful acquisition of intact sperm samples both before and after surgery, and (2) a marked improvement in total sperm motility (progressive motility (PR)+ non-progressive motility (NP)) postoperatively, defined as an absolute increase of >20 percentage points, ensuring our analysis focused on molecular changes associated with “reversible dysfunction.” Based on physical examination and color Doppler ultrasonography, all selected patients had left-sided grade II varicocele. By focusing specifically on patients who exhibited a clear improvement in semen parameters following varicocelectomy, we aimed to maximize the likelihood of identifying lactylation changes that are functionally linked to the reversible component of sperm dysfunction in varicocele. All semen samples were collected by masturbation after a standardized abstinence period of 7 days. Pre-treatment samples were collected within one week before surgery, and post-treatment samples were obtained during follow-up visits at least 3 months postoperatively. See **Table 1** for sample details.

**Table 1:** Clinical characteristics and semen parameters of the paired patient cohort before and after varicocelectomy.

Characteristics/parameters	Patient 1	Patient 2	Patient 3
Demographics			
Age (years)	32	38	36
Varicocele Grade (Left)	Grade II	Grade II	Grade II
Abstinence period (days)	7	7	7
Time after surgery (months)	3	4	3
Semen parameters (before)			
Volume (mL)	4.2	4.4	3.7
Sperm concentration (10^6^/mL)	9.5	37.5	147.3
Total sperm count (10^6^)	40.32	164.56	544.64
Total motility (PR+NP, %)	6.7	13.8	22.3
Progressive motility (PR, %)	5.8	11.6	16.4
Semen parameters (after)			
Volume (mL)	4.4	2.9	2.7
Sperm concentration (10^6^/mL)	19.4	18.2	195.5
Total sperm count (10^6^)	85.36	52.78	527.85
Total motility (PR+NP, %)	36.9	57.7	55.2
Progressive motility (PR, %)	33.3	51.8	37.7

PR, progressive motility; NP, non-progressive motility.

### Sample preparation and protein digestion

Sperm samples were initially lysed with 200 μL of lysis buffer (4% SDS, 100 mM DTT, 150 mM Tris-HCl, pH 8.0). The suspensions were heated in a boiling water bath for 3 min and subsequently subjected to ultrasonication on ice using a probe sonicator to ensure complete dissolution. Following this, any remaining undissolved cellular debris was pelleted by centrifugation at 16,000 × g for 20 min at 4°C, and the resulting supernatant was carefully collected. Protein concentration in the supernatant was determined using a BCA Protein Assay Kit.

Protein digestion was performed in accordance with the Filter-Aided Sample Preparation (FASP) protocol, as previously described by Wisniewski, Zougman et al (23). Specifically, for each sample, an aliquot containing ~100 μg of protein (based on BCA quantification) was processed. The protein extract was transferred to a 10 kDa molecular weight cut-off (MWCO) Nanosep centrifugal device (PALL, OD010C35)​ and centrifuged at 12,000 × g for 15 min. To alkylate reduced cysteine residues, 100 μL of 50 mM iodoacetamide (IAA) in UA buffer (8 M Urea, 150 mM Tris-HCl, pH 8.0)​ was added. This represents an IAA-to-protein ratio exceeding 20:1 (w/w), ensuring complete alkylation. The samples were incubated for 30 min in the dark with gentle shaking. The filter cartridge was then rinsed twice with 200 μL of UA buffer and twice with 100 μL of 100 mM ammonium bicarbonate buffer, each followed by centrifugation.

Finally, digestion was initiated by adding 40 μL of 50 mM ammonium bicarbonate buffer containing 6 μg of sequencing-grade trypsin (Promega, V5113). This constitutes a trypsin-to-protein ratio of approximately 1:16.7 (w/w). The sample was incubated at 37 °C for 16–18 hours to allow for complete digestion. After digestion, the peptides were harvested via centrifugation, acidified with 0.1% trifluoroacetic acid (TFA), and dried using a vacuum concentrator. The resulting tryptic peptides were ultimately desalted using C18 StageTips (Thermo Fisher Scientific, SP301)​ and eluted with 80% acetonitrile/0.1% formic acid prior to LC-MS/MS analysis.

### Lactylation proteomics sample preparation

Enrichment of lactylated peptides was performed via immunoaffinity purification using a commercially available, pre-conjugated pan−anti−Kla antibody−magnetic bead kit (Shanghai Baipu, China; Product Code: SHBP0618-A). According to the manufacturer, the kit is optimized for lactylome enrichment, but the specific antibody clone identifier and coupling chemistry are proprietary information. Briefly, ~100 μg of digested peptides​ (derived from the FASP preparation described above) were reconstituted in 1 mL of the provided ice−cold immunoprecipitation buffer and incubated with a pre−determined amount of the bead slurry (as per the manufacturer’s instructions for a 100 μg peptide input)​ overnight at 4 °C with gentle agitation. After incubation, the beads were collected and washed three times with 1 mL of the provided wash buffer, followed by two washes with 1 mL of LC−MS grade water. Bound peptides were then eluted by incubating the beads with 2 × 50 μL of 0.1% trifluoroacetic acid (TFA)​ for 10 minutes each. The combined eluates were dried in a vacuum concentrator. Finally, the enriched peptides were desalted and concentrated using C18 ZipTips (Millipore, ZTC18S096)​ according to the manufacturer’s protocol, eluted in 80% acetonitrile/0.1% formic acid, and dried prior to LC−MS/MS analysis.

### LC-MS analysis

LC-MS/MS were carried out on an Orbitrap Astral mass spectrometer coupled to a Vanquish Neo ultra-high-performance liquid chromatography (UHPLC) system. Each peptide sample was loaded onto a 50 cm Low-Load μPAC™ Neo HPLC column at a constant flow rate of 2.2 μL/min. The reversed-phase mobile phases consisted of (A) 0.1% formic acid in water and (B) 0.1% formic acid in 80% acetonitrile. Peptide separation was achieved using a 24-minute​ binary gradient elution at a flow rate of 1.25μL/min, with the percentage of buffer B increasing according to the following scheme: 4-6% (0-0.3 min), 6-12% (0.3-3.3 min), 12-25% (3.3-12.9 min), 25-45% (12.9-18.3 min), 45-99% (18.3-19.5 min), and held at 99% (19.5–24 min). Eluted peptides were analyzed using an Orbitrap Astral mass spectrometer. Full MS scans (survey scans) were acquired in the range of 380–980 m/z at a resolution of 240,000, with an automatic gain control (AGC) target of 500% and a maximum injection time of 5 ms. Data-independent acquisition (DIA) was performed with contiguous 2 m/z isolation windows covering 380–980 m/z (300 windows in total). The maximum injection time per window was 3 ms, with an AGC target of 500%. The normalized collision energy was set to 25%, and the cycle time was 0.6 s. Full MS spectra were recorded in profile mode, and DIA MS/MS spectra were acquired in centroid mode. The full MS scan range for the instrument was 150–2000 m/z.

### Database searching and analysis

The DIA-MS data were analyzed using Spectronaut 18 (Biognosys AG, Switzerland). The MS data were searched against the UniProtKB Homo sapiens database [9606]-205104-20241204.fasta (205,104 entries, downloaded on 2024-12-04; available at: https://www.uniprot.org/taxonomy/9606). Tryptic cleavage specificity was applied, along with variable methionine oxidation (M), variable protein N-terminal acetylation, variable Lactylation on (K) and fixed carbamidomethyl cysteine modifications. The minimum 6 amino acids for peptide, and at least 1 unique peptide were required per protein. For peptide and protein identification, false discovery rate (FDR) was set to 1%. Site quantitation analysis was filtered only for those lactylation​ sites that were confidently localized >=0.75 site probability with algorithm. Label-free quantification was carried out using intensity determination. The quantitative site ratios were weighted and normalized by the median ratio.

### Bioinformatics analysis

Bioinformatic analyses were performed using Perseus, Microsoft Excel, Python, and the R statistical computing environment. Missing values were imputed and datasets were resampled using the K-Nearest Neighbors (KNN) algorithm. Significantly differentially expressed sites were identified based on the thresholds of a fold change >2 or <0.5 and an FDR-adjusted P-value < 0.05. Expression data were subjected to hierarchical clustering at the site level using the open-source R language (v25), with Euclidean distance as the metric and the complete linkage method for agglomeration. Functional annotation of sequences was retrieved from UniProtKB/Swiss-Prot, the Kyoto Encyclopedia of Genes and Genomes (KEGG), and the Gene Ontology (GO) database. Enrichment analyses for GO and KEGG terms were conducted using Fisher’s exact test, with FDR correction for multiple comparisons. GO terms were categorized into biological process (BP), molecular function (MF), and cellular component (CC). Terms and pathways with a corrected p-value below 0.05 were considered statistically significant. Protein–protein interaction (PPI) networks were constructed using the STRING database and visualized in Cytoscape. Additionally, significantly enriched lactylation​ motifs for kinase-substrate interactions were identified with the MOMO software tool.

### Protein extraction

Sperm protein extracts were prepared from normozoospermic and asthenozoospermic samples. Briefly, frozen sperm samples were rapidly thawed on ice and homogenized in ice-cold RIPA lysis buffer supplemented with protease inhibitor cocktail (1%, v/v) and 5 mM dithiothreitol. The homogenates were subjected to ultrasonic disruption on ice using a probe sonicator (3 cycles of 5-s pulses at 200 W with 10-s intervals). The resulting lysates were then centrifuged at 12,000 ×g for 15 min at 4 °C to pellet insoluble debris. The clarified supernatants were collected, and protein concentration was determined using a BCA Protein Assay Kit (Bio-Rad, USA) according to the manufacturer’s instructions. Aliquots of the protein extracts were stored at –80 °C until further analysis.

### Immunoprecipitation and western blot analysis​

To validate the lactylation abundance of key motility-related proteins identified in our lactylome analysis, immunoprecipitation (IP) followed by western blotting (WB) was performed. The target proteins included PGK2, TEKT3, AKAP4, TUBA1A, AKAP3, and TUBB4B. Sperm protein lysates from both normal and asthenozoospermic samples were prepared using RIPA lysis buffer supplemented with protease and phosphatase inhibitors. The protein concentration was determined using a BCA Protein Assay Kit. For each immunoprecipitation reaction, 20 mg of total protein lysate was incubated with specific primary antibodies against the target proteins (PGK2, TEKT3, AKAP4, TUBA1A, AKAP3, and TUBB4B purchased from Proteintech, China) that had been pre-conjugated to Protein A/G Magnetic Beads (Beyotime, China). The mixture was incubated with gentle rotation overnight at 4 °C. After incubation, the beads were washed extensively with ice-cold wash buffer to remove non-specifically bound proteins. The immunoprecipitated complexes were then eluted by boiling in 2× SDS loading buffer. The eluted proteins were separated by SDS-PAGE and transferred onto PVDF membranes. The membranes were blocked with 5% non-fat milk and subsequently incubated with an anti-L-Lactyl Lysine Rabbit mAb-PTM1401RM (PTM Bio, China; 1:1000 dilution) overnight at 4 °C to detect the total lactylation abundance of the target proteins. After washing, the membranes were incubated with HRP-conjugated secondary antibodies (1:10,000 dilution; Affinity, China) and the signals were visualized using an enhanced chemiluminescence (ECL) detection system (MeiLunBio, China).

### Immunofluorescence staining of sperm lactylation​

To assess the global lactylation abundance and subcellular localization in spermatozoa, immunofluorescence staining was performed on samples from both normozoospermic and asthenozoospermic groups. Approximately 2×10^6^ sperm cells were resuspended in 1 mL of phosphate-buffered saline (PBS). A 10 μL aliquot of this suspension was evenly spread onto a glass slide and air-dried. Slides were fixed in 4% paraformaldehyde (PFA) for 30 minutes at room temperature and subsequently washed three times with PBS (10 minutes per wash). Non-specific binding sites were blocked by incubating the slides with 5% bovine serum albumin (BSA) in PBS for 1 hour at room temperature. The slides were then incubated overnight at 4 °C with an anti-L-Lactyl Lysine Rabbit mAb, diluted at 1:100 in PBS containing 1% BSA.Following primary antibody incubation, the slides were thoroughly washed three times with PBS (10 minutes each) and then incubated with an Alexa Fluor^®^ 488-conjugated goat anti-rabbit IgG secondary antibody (Invitrogen, USA) for 2 hours at room temperature in the dark. After additional PBS washes, nuclear DNA was counterstained with 2 μg/mL 4′,6-diamidino-2-phenylindole (DAPI; Sigma-Aldrich, USA) for 20 minutes. Finally, the slides were washed again, mounted with anti-fade mounting medium, and visualized under a fluorescence microscope. Image acquisition and scale calibration were performed using ImageJ software (NIH, USA).

### Measurement of sperm L-lactate levels​

The intracellular L-lactate concentration in spermatozoa from normozoospermic and asthenozoospermic individuals was quantified using a commercial L-Lactate Assay Kit (WST-8 method, Beyotime Biotechnology, China) according to the manufacturer’s instructions. Briefly, sperm cells were lysed with the provided BeyoLysis™ Buffer A on ice. The lysates were centrifuged at 12,000 × g for 10 min at 4 °C to remove insoluble debris. The supernatant was collected and incubated with the WST-8 working solution, which contains L-lactate dehydrogenase, NAD+, and the colorimetric probe WST-8. The reaction mixture was incubated at 37 °C for 30 minutes in the dark, during which L-lactate was enzymatically converted, leading to the reduction of WST-8 to a formazan product proportional to the lactate concentration. The absorbance was measured at 450 nm using a microplate reader. A standard curve generated with known concentrations of L-lactate was used to calculate the precise lactate concentration in each sample, which was normalized to the total protein content determined by a BCA assay.

### Statistical analysis

Data are presented as mean ± standard deviation (SD). The normality of data distribution was assessed using the Shapiro-Wilk test. Statistical comparisons between the asthenozoospermia and normozoospermia groups were performed using an unpaired two-tailed Student’s t-test for normally distributed data or the Mann-Whitney U test for non-normally distributed data. A P-value of less than 0.05 was considered statistically significant. All statistical analyses for these experiments were performed using GraphPad Prism (version 9.0.0).

## Results

### Varicocele-associated asthenozoospermia is characterized by disrupted lactate metabolism and downregulated protein lactylation in sperm

**Figure 1:**
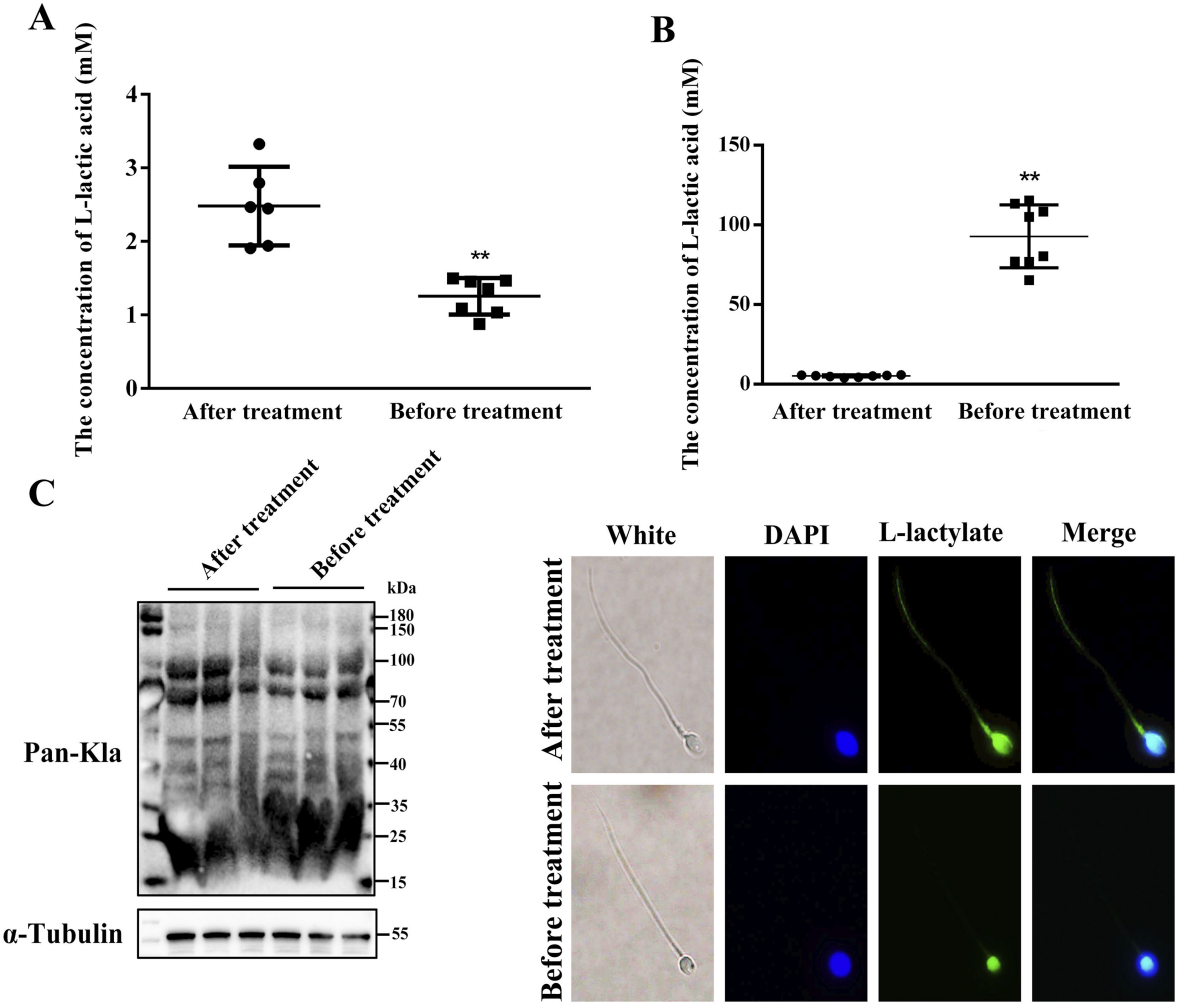
Changes in lactate metabolism and protein lactylation in spermatozoa from varicocele-associated asthenozoospermia before and after treatment. **(A)** Measurement of intracellular L-lactate levels in spermatozoa (n = 6).​ Compared to the post-treatment group, the intracellular L-lactate level was significantly decreased in sperm from the pre-treatment group (**p < 0.01). Statistical analysis was performed using the Student’s t-test. **(B)** Measurement of L-lactate concentration in seminal plasma (n = 8).​ The L-lactate level in seminal plasma was significantly elevated in the pre-treatment asthenospermia group (**p < 0.01). Statistical analysis was performed using the Student’s t-test. **(C)** Western blot analysis of total protein lactylation in spermatozoa.​ Pan-Kla antibody showed decreased overall lactylation band intensity in the asthenospermia group, with α-Tubulin serving as the loading control (n = 3). **(D)** Immunofluorescence staining of spermatozoa.​ Pan-Kla (green) indicates that lactylated proteins are primarily localized in the sperm midpiece and principal piece; DAPI (blue) stains the nucleus. Compared to the After treatment, the pan-lactylation signal was markedly weaker in the Before treatment group. Scale bar = 20 μm. Statistical analysis was performed using the Student’s t-test.

Semen samples were collected from patients with varicocele-associated asthenozoospermia before and after treatment. Analysis of L-lactate levels in spermatozoa and seminal plasma revealed that compared to post-treatment normozoospermic samples, the intracellular L-lactate content was significantly decreased in spermatozoa from the pre-treatment varicocele-associated asthenozoospermia group (**Figure 1A**). In contrast, the concentration of L-lactate in the seminal plasma was significantly elevated (**Figure 1B**), suggesting impaired lactate uptake or retention capacity and increased lactate efflux in asthenozoospermic sperm. Further investigation using a pan-lactylation antibody revealing an altered profile of lysine lactylation in sperm total proteins from the pre-treatment varicocele-associated asthenozoospermia samples. This qualitative observation suggests that the global lactylation pattern is disrupted (**Figure 1C**). Immunofluorescence results also indicated a marked decrease in pan-lactylation fluorescence signals in varicocele-associated asthenozoospermia sperm, predominantly localized in the midpiece and tail regions (**Figure 1D**; **Supplementary Figure S1**), indicating that lactylation modification was significantly reduced in functional domains of the flagellum. In summary, varicocele-associated asthenozoospermia is accompanied by dysregulated lactate metabolism and a significant downregulation of protein lactylation in sperm.

### Global characterization of the sperm lactylome reveals cytoplasmic enrichment and key pathways in motility regulation​

**Figure 2:**
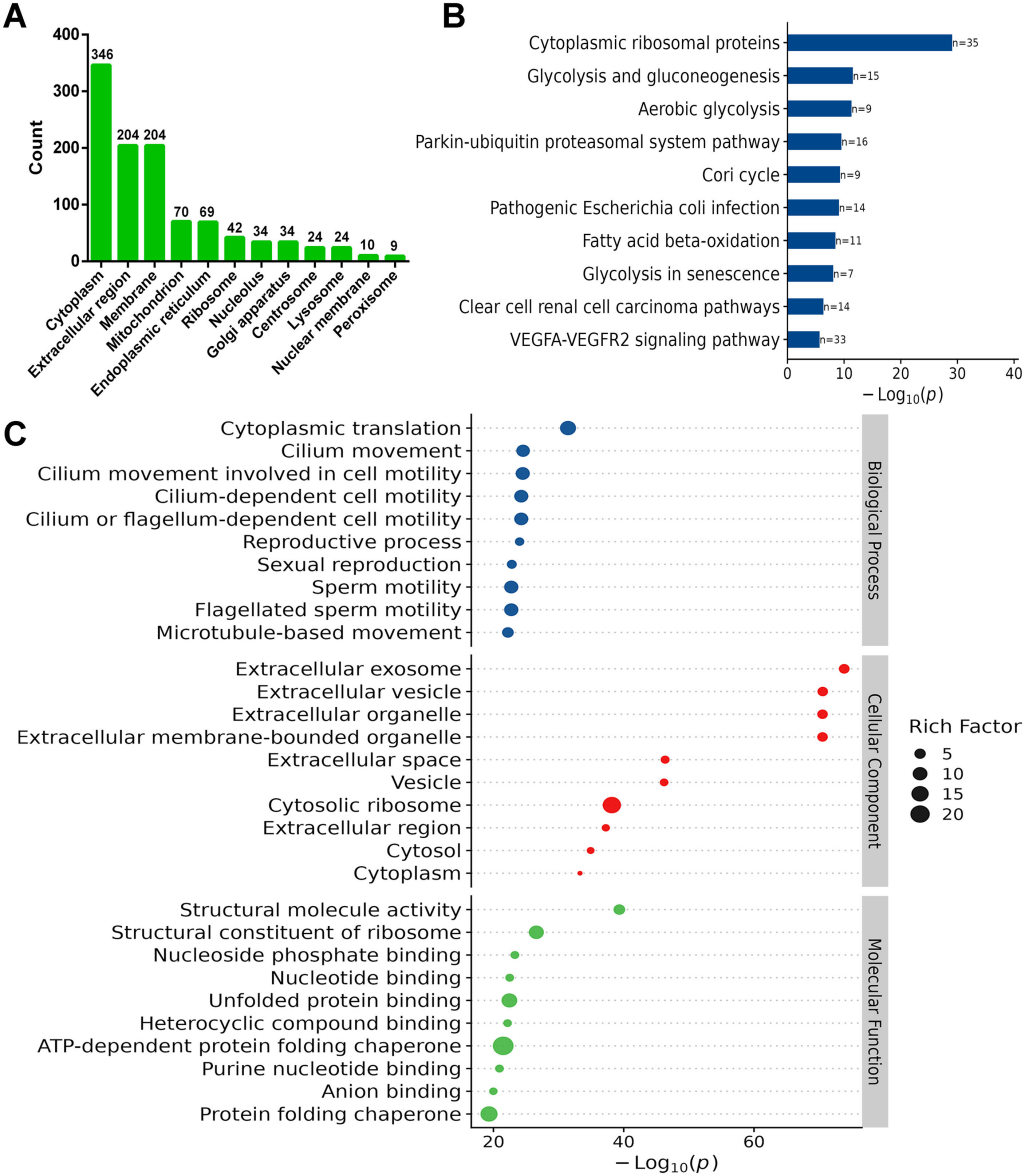
Subcellular localization and functional enrichment analysis of lactylated proteins in sperm from asthenozoospermic and normal control individuals. **(A)**​​ Subcellular localization annotation of all identified lactylated proteins based on the GO-Cellular Component database. **(B)** Top 10 enriched terms from Wiki Pathways analysis. **(C)** GO enrichment analysis.

To comprehensively elucidate protein lactylation in asthenozoospermic sperm, we conducted a proteome-wide lactylome analysis of sperm from individuals with asthenozoospermia. Lactylated peptides from proteins in each group were enriched using a pan-anti-Kla antibody and analyzed by LC–MS/MS. The initial database search identified spectral matches corresponding to 2,699 lactylation sites on 1,216 protein entries​ (**Supplementary Tables 1, S2**). To obtain a non-redundant set reflecting unique biological entities, entries mapping to the same gene were consolidated.​ This refinement yielded a core dataset of 831 unique lysine lactylation sites on 405 distinct proteins​ (**Supplementary Tables 3, S4**), corresponding to an average of 2.05 sites per protein. The distribution of lactylation sites among proteins was notably uneven​ (**Supplementary Figure S2A**). All high-confidence identified lactylated proteins were annotated using the GO-Cellular Component database, yielding 1071 localization entries spanning 13 subcellular compartments. Consistent results from bar/pie/bubble charts (**Figure 2A**) revealed the cytoplasm as the most enriched compartment, containing 346 proteins (32.34%), followed by the extracellular region (204 proteins, 19.07%) and the membrane system (204 proteins, 19.07%). In contrast, structures such as the centrosome, lysosome, nuclear membrane, and peroxisome accounted for relatively low proportions (each <3%). Further WikiPathways enrichment (**Figure 2B**) and GO-CC functional clustering (**Figure 2C**) of all lactylated proteins identified significant enrichment of terms including “cytoplasmic translation,” “glycolysis/gluconeogenesis,” “fatty acid β-oxidation,” “Parkin-ubiquitin proteasome system,” “VEGFA-VEGFR2 signaling pathway,” and “microtubule-based movement.” Notably, processes such as “cytoplasmic ribosomal proteins,” “flagellar movement,” “sperm motility,” and “extracellular vesicles” were collectively highlighted, suggesting that lactylation may intricately regulate sperm motility by synchronously modulating cytoplasmic energy metabolism, protein homeostasis, and flagellar microtubule function. These results provide a reliable subcellular localization and pathway framework for subsequent screening of differential sites and functional validation.

### Differential lactylation profiling in asthenozoospermic sperm reveals aberrant modifications in flagellar architecture and energy metabolism

**Figure 3:**
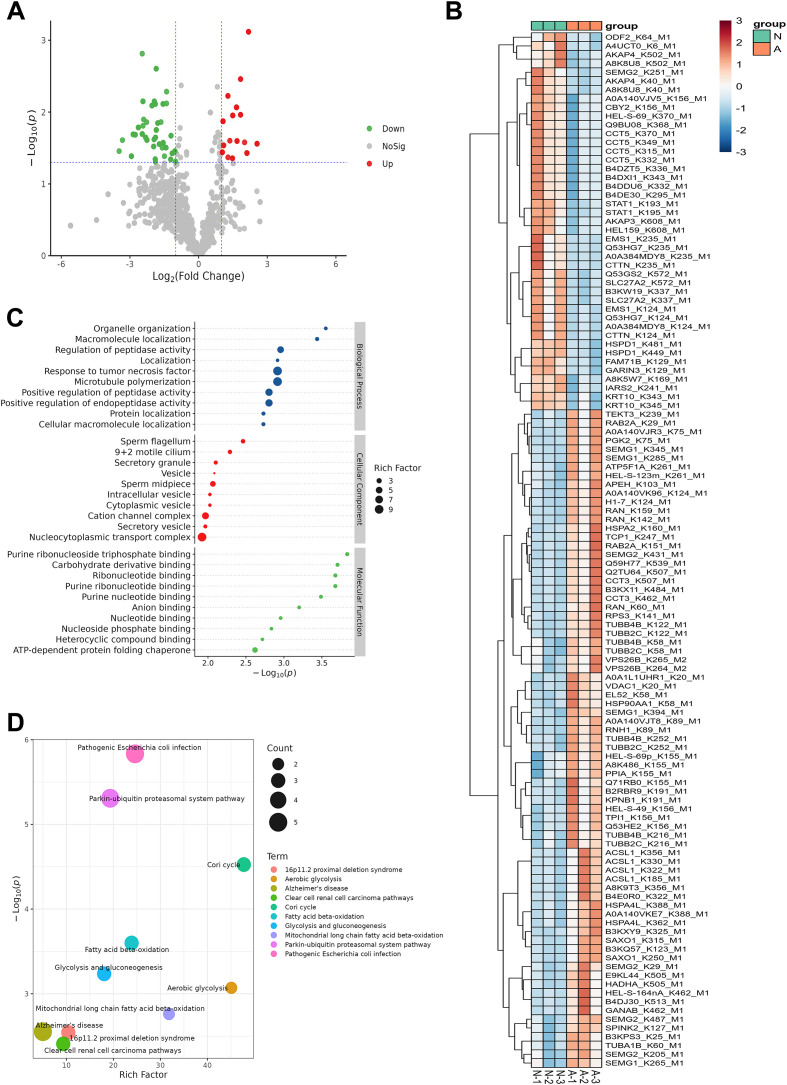
Comprehensive analysis of expression profiles and functional enrichment of differentially lactylated proteins and sites. **(A)** Volcano plot displaying differentially lactylated proteins. **(B)** Heatmap illustrating differentially lactylated proteins. **(C)** GO enrichment analysis. **(D)** Top 10 enriched terms from WikiPathways analysis.

To identify differential protein lactylation between asthenozoospermic and control sperm, we performed an analysis using thresholds of |log_2_FC| ≥ 1 and p < 0.05. This yielded 133 differentially lactylated sites, corresponding to 78 up-regulated and 55 down-regulated modification events (**Figures 3A, B**; **Supplementary Table 5**). After consolidating multiple lactylation sites from the same protein, we obtained a non-redundant set of 85 differentially lactylated proteins​ (**Supplementary Table 6**). GO enrichment analysis revealed that these differential proteins were significantly associated with biological processes such as “macromolecule localization,” “organelle organization,” “protein localization,” “microtubule polymerization,” and “positive regulation of peptidase activity.” Regarding cellular components, the differentially modified proteins were predominantly localized to the “sperm flagellum,” “9 + 2 motile cilium,” “sperm midpiece,” and various vesicle structures, suggesting that aberrant lactylation events are highly concentrated in flagellar microtubule and energy metabolism-related compartments. In terms of molecular function, the proteins were significantly enriched in “purine nucleotide binding,” “nucleoside phosphate binding,” and “ATP-dependent protein folding chaperone” activities (**Figure 3C**). WikiPathways enrichment analysis revealed that the differentially lactylated proteins were significantly enriched in multiple pathways (**Figure 3D**). Among these, the most significantly enriched pathways included ‘Pathogenic Escherichia coli infection’, ‘Parkin-ubiquitin proteasomal system pathway’, and ‘Cori cycle’. Concurrently, metabolic pathways relevant to the focus of this study, such as ‘glycolysis/gluconeogenesis’ and ‘Fatty acid beta-oxidation’, also showed significant enrichment.

### Protein functional interaction network analysis of proteins containing differential lactylation sites

**Figure 4:**
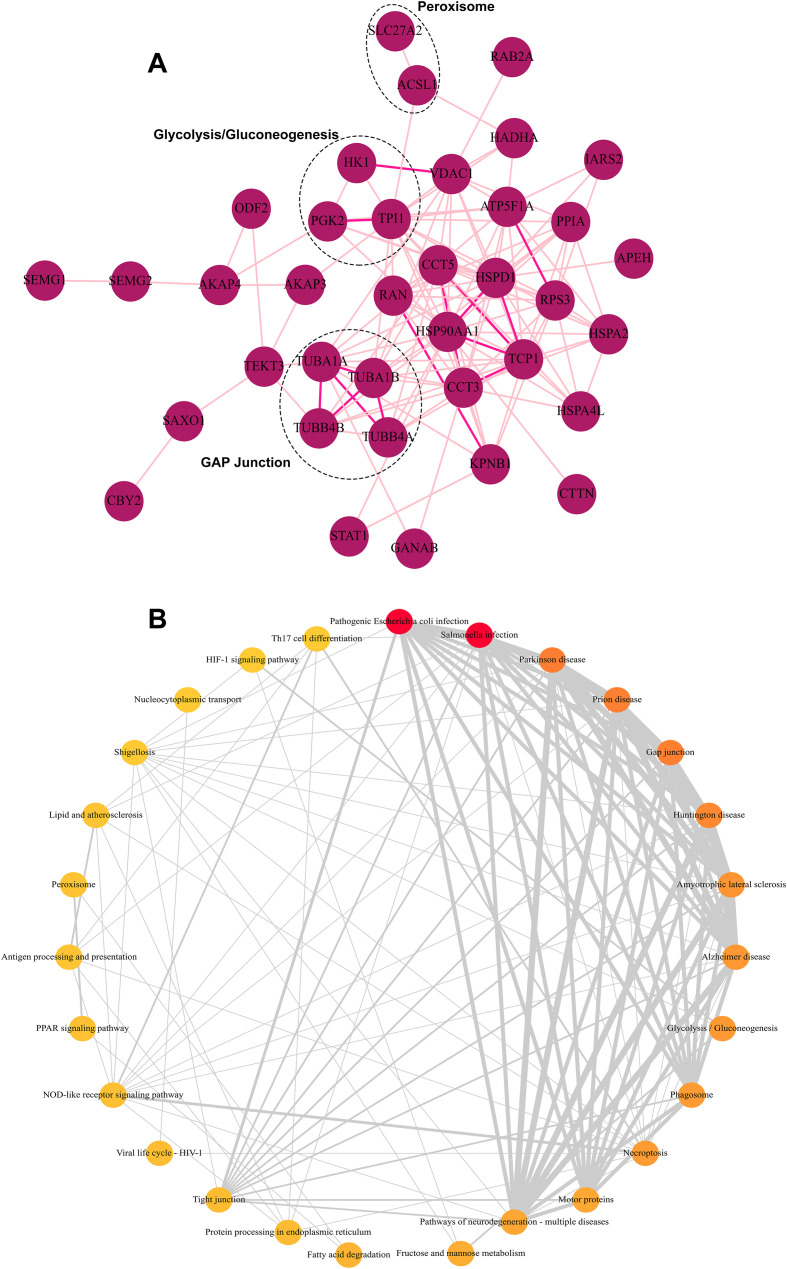
Interaction network and enriched pathway information of differentially lactylated proteins. **(A)** Protein functional interaction Network (PFIN) analysis using the STRING database, visualized with top 50 hub nodes identified by the PageRank algorithm. **(B)** Protein-protein interaction network of the top 50 hub nodes, overlaid with enriched pathway information.

To gain deeper insights into the cellular processes through which differentially lactylated proteins regulate asthenozoospermia, we constructed an integrated network by combining protein–protein interaction data from the STRING database with pathway–protein associations. Furthermore, the top 50 hub nodes were identified using the PageRank algorithm and utilized for network construction (**Supplementary Table 7**). Our analysis revealed that several protein complexes—including the peroxisome complex, gap junction complex, and glycolysis/gluconeogenesis complex—underwent actylation, suggesting that these differentially lactylated complexes may be implicated in the etiology of asthenozoospermia (**Figure 4**). Subsequent modification site analysis demonstrated that core members of these complexes, such as SLC27A2, HK1, PGK2, ACSL1, TPI1, TUBA1A, TUBA1B, TUBB4A, and TUBB4B, collectively contained 50 lactylation sites (**Supplementary Table 8**). These findings indicate that these extensive lactylation modifications may play crucial roles in regulating gene expression in individuals with asthenozoospermia. In conclusion, our results suggest that lactylation-mediated protein interactions among these complexes are likely involved in modulating the pathological progression of asthenozoospermia.

### ​​Identification of a lactylation-regulated protein signature associated with sperm motility​

**Figure 5:**
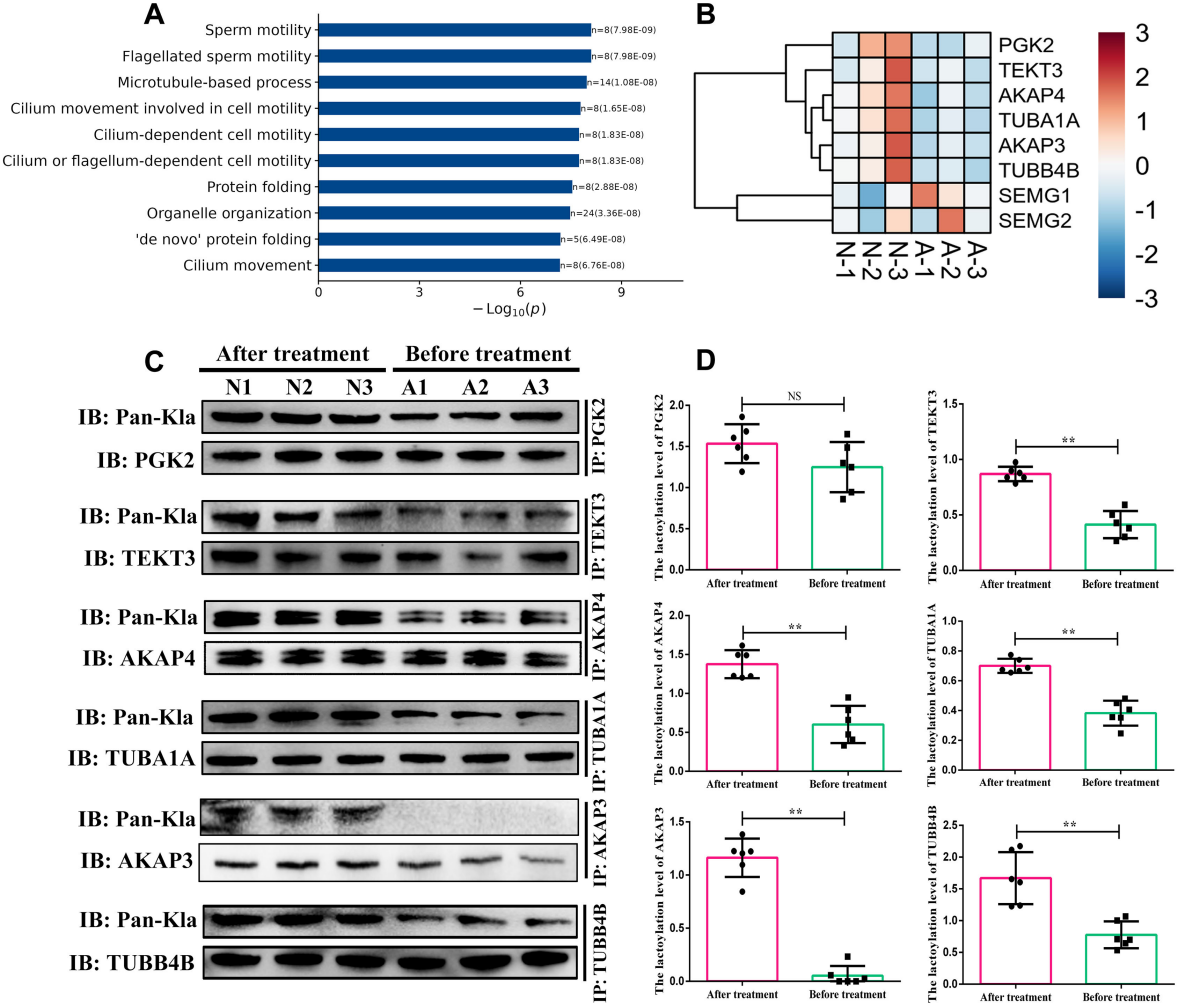
Lactylation-regulated signature proteins associated with sperm motility. **(A)** GO enrichment analysis of biological processes. **(B)** Heatmap of differentially lactylated proteins. **(C, D)** Western blot analysis was performed to validate the lactylation abundance of proteins in sperm from patients with asthenozoospermia caused by varicocele, both before and after treatment. Pan‐Kla antibody was used to detect total protein lactylation. Before treatment, the lactylation signals of TEKT3, AKAP4, TUBA1A, AKAP3, and TUBB4B were significantly attenuated, while PGK2 showed a decreasing trend that did not reach statistical significance. After treatment, the lactylation abundance of these proteins were partially restored. Data are presented as mean ± SEM (n = 3). **p < 0.01, NS, not significant. Statistical analysis was performed using the Student’s t-test.

Given that diminished energy metabolism and motility are direct causes of asthenozoospermia (5, 24), our GO enrichment analysis of the lactylated proteome revealed “flagellated sperm motility” and “sperm motility” as the most significantly enriched terms under the Biological Process category. These terms were associated with the same set of eight lactylated proteins: PGK2, TEKT3, AKAP4, TUBA1A, AKAP3, TUBB4B, SEMG1, and SEMG2 (**Figure 5A**). Heatmap analysis of these eight differentially lactylated proteins demonstrated that the abundance of PGK2, TEKT3, AKAP4, TUBA1A, AKAP3, and TUBB4B were significantly decreased in asthenozoospermic sperm, whereas the levels of SEMG1 and SEMG2 were markedly increased (**Figure 5B**). Since the total protein levels of SEMG1 and SEMG2 were also significantly elevated in asthenozoospermia, whereas the expression of PGK2, AKAP4, TUBA1A, AKAP3, and TUBB4B remained unchanged, and TEKT3 expression was increased (**Supplementary Figure S2B**), this suggests that the increase in lactylation modification of SEMG1 and SEMG2 may be attributable to their elevated total protein abundance. We therefore focused on assessing the lactylation abundance of the remaining six proteins (PGK2, TEKT3, AKAP4, TUBA1A, AKAP3, and TUBB4B), in which lactylation changes appeared independent of total protein abundance. The specific lysine lactylation sites identified for each of these candidate proteins are detailed in **Table 2**. The results showed that the lactylation abundance of TEKT3, AKAP4, TUBA1A, AKAP3, and TUBB4B proteins were significantly decreased in sperm from patients with asthenozoospermia caused by varicocele before treatment, while the lactylation level of PGK2 also exhibited a downward trend, although this change was not statistically significant (**Figures 5C, D**).

**Table 2:** Lactylation sites of candidate proteins identified in sperm.

Gene name	Protein descriptions	Site location (K)	Number of location
*PGK2*	Phosphoglycerate kinase 2	30, 48, 75, 97, 106, 141, 216, 353, 361, 365	10
*TEKT3*	Tektin-3	239	1
*AKAP4*	A kinase (PRKA) anchor protein 4	40, 92, 137, 139, 234, 245, 258, 269, 299, 323, 353, 359, 368, 376, 408, 428, 439, 442, 452, 471, 488, 495, 502, 511, 532, 542, 550, 594, 599, 623, 662, 803, 810, 814	34
*TUBA1A*	Tubulin alpha-1A chain	25, 61, 291, 301, 335, 359, 366	7
*AKAP3*	A-kinase anchor protein 3	51, 62, 173, 213, 309, 327, 369, 396, 400, 410, 425, 447, 455, 608, 616	15
*TUBB4B*	Tubulin beta-4B chain	58, 122, 216, 252, 297, 324, 350, 362	8

## Discussion

The landscape of protein lactylation in sperm is beginning to be unveiled. A recent pioneering study by Yan et al. (2025) provided the first lactylomic profile of human sperm, identifying 220 lactylated proteins and establishing a functional link between hypo-lactylation of the glycolytic enzyme PGK2 and impaired sperm motility in idiopathic asthenozoospermia (17). This work confirms the existence and functional relevance of this modification in the male gamete. Our study builds upon this foundation by asking an etiology-specific question: how is the sperm lactylome altered in the context of a prevalent and treatable condition—varicocele—and does it dynamically respond to corrective surgery?​ Using a paired pre- and post-treatment design, our study provides observational evidence linking dynamic changes in the sperm lactylome to the recovery of sperm motility following varicocelectomy. Our analysis successfully mapped the landscape of protein lactylation, identifying 831 lactylation sites across 405 proteins. Notably, we detected 133 differentially lactylated sites between the asthenozoospermic and control groups, with 78 down-regulated and 55 up-regulated. These altered proteins were significantly enriched in key biological processes, including sperm flagellar motility and pathways such as glycolysis/gluconeogenesis. These findings not only highlight alterations in protein lactylation associated with asthenozoospermia, but also provide a novel perspective and candidate mechanisms for understanding how varicocele may impair sperm quality. The effect of lactic acid on sperm motility has been confirmed in studies of various animal species (25–29). Although seemingly contradictory results exist among different species (25, 30), lactic acid remains a key player in regulating sperm energy metabolism. Metabolomic studies on sperm from asthenozoospermia caused by varicocele have revealed a significant decrease in sperm lactic acid levels (6), which is consistent with our detection results and also aligns with findings in obesity-associated asthenozoospermia (31). Furthermore, we found a significant increase in L-lactic acid levels in the seminal plasma of patients with asthenozoospermia caused by varicocele, which is also consistent with recent clinical research finding (32). As a key metabolic intermediate, L-lactic acid not only plays an important role in energy metabolism but also influences cell signaling and gene expression through lactylation (13). The total abundance of lactylated proteins were decreased in sperm from patients with asthenozoospermia caused by varicocele, with the most pronounced reduction observed in the midpiece and tail regions, suggesting that protein lactylation may be involved in regulating sperm flagellar movement.

Functional enrichment analysis of differentially lactylated proteins in varicocele-associated asthenozoospermia revealed distinct molecular characteristics. GO analysis demonstrated that these proteins were significantly enriched in terms directly pertinent to sperm structure and function. Key enrichments in the Cellular Component​ category included ‘Sperm flagellum’and ‘9 + 2 motile cilium’, pinpointing the flagellar apparatus as a major site of lactylation alteration. Concurrently, enrichments in Molecular Function, such as ‘Purine ribonucleoside triphosphate binding’ and ‘ATP-dependent protein folding chaperone’, along with Biological Processes​ like ‘Microtubule polymerization’ and ‘Organelle organization’, strongly implicate lactylation in the regulation of energy metabolism​ and cytoskeletal dynamics​ essential for motility. Our WikiPathways analysis revealed an additional and intriguing layer:​ among the most significantly enriched pathways were several associated with human diseases, such as ‘Pathogenic Escherichia coli infection’, ‘Parkin-ubiquitin proteasomal system pathway’, ‘Alzheimer’s disease’, and ‘Clear cell renal cell carcinoma pathways’. This observation likely reflects an inherent bias in current protein function annotation databases, where many proteins are primarily studied and annotated within disease contexts. Therefore, while these enrichments are statistically prominent, their biological interpretation in the context of sperm requires caution. Crucially, and consistent with our hypothesis, pathways with clear relevance to sperm biology were also significantly enriched.​ These included ‘glycolysis/gluconeogenesis’ and ‘Fatty acid beta-oxidation’, which are central to energy production in spermatozoa. The co-enrichment of these metabolic pathways with flagellar structural terms from the GO analysis provides convergent evidence that the dysregulated lactylation in varicocele may concurrently disrupt both the energetic supply​ and the structural integrity​ necessary for effective sperm movement. An intriguing aspect of our data is the heterogeneous distribution of lactylation sites across proteins.​ The majority of modified proteins (280 proteins, 33.6%) harbored only a single lactylation site, suggesting a role for this modification in fine-tuned, specific regulation. In contrast, a subset of proteins underwent hyper-lactylation, with AKAP4 and SEMG1 bearing 34 and 21 identified sites, respectively. This non-uniform distribution is consistent with previous lactylome studies in other systems and underscores a potential hierarchy in lactylation-mediated regulation (33). Proteins with multiple modification sites may undergo allosteric or multimodal tuning​ of their function, a phenomenon observed with other dense post-translational modifications (34). Among our six motility-related candidate proteins, AKAP4 and AKAP3 stood out with 34 and 15 potential lactylation sites, respectively, indicating that they might be central hubs for lactate-mediated signaling​ with the potential for multi-faceted functional regulation. The specific mechanisms and functional outcomes of this extensive modification on AKAP4 and AKAP3 warrant dedicated future investigation.

Lactic acid plays a dual role in sperm biology: as a metabolic regulator (35) and a potential motility modulator (25). In sperm, it is actively produced and may influence the intracellular milieu, thereby affecting flagellar function (36). The core of sperm motility relies on efficient ATP production via glycolysis and oxidative phosphorylation. In asthenozoospermia, this energy metabolism is frequently compromised, leading to reduced motility (37, 38). Our results indicate that the differentially lactylated proteins identified in sperm from varicocele-associated asthenozoospermia were predominantly enriched in processes such as glycolysis/gluconeogenesis, sperm flagellum, and ATP-dependent protein folding chaperone. These findings suggest that the maintenance of normal sperm motility may be heavily reliant on the lactylation modification of proteins associated with energy metabolism and sperm structural integrity.

One particularly finding of our study is that differentially lactylated proteins are highly enriched in sperm-specific cellular structures such as the flagellum and the fibrous sheath. Specifically, we observed significant alterations in lactylation of tubulins (TUBA1A, TUBA1B, TUBB4B) and fibrous sheath structural proteins (AKAP3, AKAP4, TEKT3). Microtubules form the core components of the flagellar axoneme, while the fibrous sheath serves as a critical signaling scaffold and structural platform in the sperm tail. The integrity of these structures is essential for flagellar movement. Microtubules, as the central elements of the axoneme, exhibit a high degree of structural and functional conservation across species. Studies have shown that flagellar motility relies on a microtubule sliding mechanism, which is driven by ATP-powered motor proteins such as dynein (39, 40). AKAP3 and AKAP4 are the major structural proteins of the sperm fibrous sheath and are essential for sperm motility and flagellar formation. Studies have shown that reduced expression of *AKAP4* leads to impaired sperm motility and is associated with male infertility (41, 42). Furthermore, the structural stability of AKAP3 significantly influences sperm movement, and certain mutations may lead to functional diversity, thereby affecting male fertility (43). TEKT3 is another important protein associated with sperm motility. Research indicates that its expression in the sperm axoneme is critical for maintaining normal sperm motility and morphology. Deficiency or loss of function of *TEKT3* results in reduced sperm motility and may contribute to male infertility (44). Moreover, TEKT3 interacts with other tektin proteins to form complexes, and this interaction is vital for normal sperm function (44). Tubulins such as TUBA1A, TUBA1B, and TUBB4B also play important roles in the formation and movement of the sperm flagellum. Studies suggest that the expression levels of these proteins are closely correlated with sperm motility, particularly in sperm following cryopreservation and thawing (45). Therefore, altered lactylation of these structural proteins may directly affect the assembly and stability of the flagellum. In addition, our PFIN analysis suggested that these lactylated proteins may function within specific complexes. The peroxisome complex, gap junction complex, and glycolysis/gluconeogenesis complex were identified as potentially key complexes through which lactylated proteins participate in regulating sperm function in varicocele-associated asthenozoospermia. Finally, Western blot analysis successfully confirmed a significant reduction in lactylation abundance of five core proteins—TEKT3, AKAP4, TUBA1A, AKAP3, and TUBB4B—which are highly relevant to “glycolysis” and “sperm motility,” in asthenozoospermic sperm. Collectively, these findings are consistent with a novel perspective wherein alterations in protein lactylation are associated with concurrent disturbances in both the”energy supply” and the”motility apparatus” of sperm, potentially contributing to the pathogenesis of asthenozoospermia.

Our study, focusing on varicocele-associated asthenozoospermia, reveals dynamic reprogramming of sperm protein lactylation following surgical intervention. This finding aligns with and extends the recent work by Yan et al., who conducted a comprehensive lactylome analysis on a general asthenozoospermia cohort and identified 220 lactylated proteins, with significant enrichment in glycolytic pathways and sperm motility-related functions. Notably, both studies converge on the central role of PGK2. Yan et al (17). reported that PGK2 is lactylated at 10 lysine residues and that its lactylation level and enzymatic activity are significantly downregulated in asthenozoospermia sperm, leading to impaired ATP production and motility. They further demonstrated that exogenous supplementation of PGK2 downstream products could partially rescue sperm motility defects. The overlap in identifying PGK2 lactylation deficiency​ as a key feature suggests that dysregulation of glycolytic enzyme activity via lactylation may represent a common pathogenic pathway in asthenozoospermia, irrespective of the specific etiology. However, our varicocele-specific longitudinal design also uncovered lactylation alterations on proteins associated with oxidative stress response and heat-shock pathways, which were not highlighted in the general asthenozoospermia cohort. Furthermore, we identified significant hypo-lactylation of key flagellar structural components like AKAP4 and TEKT3. These unique alterations may point to etiology-specific mechanisms​ linked to the distinct pathophysiological microenvironment of varicocele, such as local hyperthermia, oxidative stress, and consequent structural vulnerability of the sperm flagellum. Therefore, while our work corroborates the fundamental importance of protein lactylation, particularly on metabolic enzymes like PGK2, in male subfertility, it also delineates a subset of modifications that could be specific to varicocele, offering potential targets for more precise diagnostic or therapeutic strategies.

## ​​Conclusion

In summary, this study presents a comprehensive lactylomic profile of human spermatozoa, revealing significant alterations in the lactate-lactylation axis associated with varicocele-associated asthenozoospermia. We observed that this condition is characterized by concurrent decreases in intracellular lactate, elevated seminal plasma lactate, and a global reduction in protein lactylation. Integrative analysis identified hypo-lactylation of key flagellar structural and metabolic proteins, including AKAP4, AKAP3, TEKT3, and glycolytic enzymes. These associations support a model in which dysregulated lactylation could concurrently impair both the structural integrity of the sperm motility apparatus and its localized energy supply. Our findings highlight protein lactylation as a metabolite-associated modification in human sperm and correlate its dysregulation with a common form of male infertility, providing a novel framework for future mechanistic exploration.

### Limitations and future research directions

It is important to note the primary limitation of this work: the relatively small cohort size.​ While the paired pre- and post-treatment design strengthens internal validity, the limited sample size constrains statistical power and generalizability. Furthermore, the other limitation of this study is the absence of a control group consisting of individuals with asthenozoospermia due to non-varicocele etiologies.​ While our paired pre- and post-treatment design effectively captures treatment-associated dynamics within the same individual, it cannot determine whether the observed alterations in the lactate-lactylation axis are specific to varicocele or represent a common pathway in idiopathic asthenozoospermia. Therefore, the inclusion of such a control group in future studies will be essential to establish the etiological specificity of the protein lactylation signature identified here. The findings, particularly the identification of specific differentially lactylated proteins, should be considered preliminary and require validation in larger, independent cohorts. All interpretations are necessarily tempered by the exploratory nature and sample size of this study.

Within these constraints, our results collectively suggest a potential role for the lactate-lactylation axis​ in concurrently impairing both the energy supply and structural apparatus required for sperm motility. This work thus offers a novel perspective on the molecular pathogenesis of varicocele-associated male infertility and lays the groundwork for future studies​ to explore the functional and translational significance of the identified lactylation targets. Future research should focus on functionally validating these targets in sperm motility and investigating their potential utility.

## Data Availability

The mass spectrometry proteomics data have been deposited to the ProteomeXchange Consortium via the PRIDE partner repository with the data set identifier PXD074818 (https://www.iprox.cn/page/PDV0141.html).
