# Supplementary material for: Alterations in the protein lactylation landscape of sperm from patients with varicocele-associated asthenozoospermia
Source: Front Endocrinol (Lausanne). 2026 Jun 23;17:1791920. doi: 10.3389/fendo.2026.1791920 (PMC13337366; doi:10.3389/fendo.2026.1791920)
Supplement: Supplementary file 9 [file DataSheet1.pdf]

Figure 1

The sequence of protein loading was as follows:

Control-1, Control-2, Control-3, Asthenospermia-1, Asthenospermia-2, Asthenospermia-3.

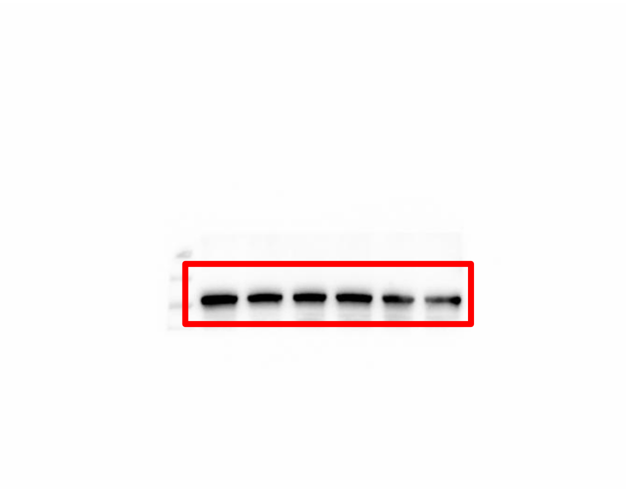

α-Tubulin 55kDa

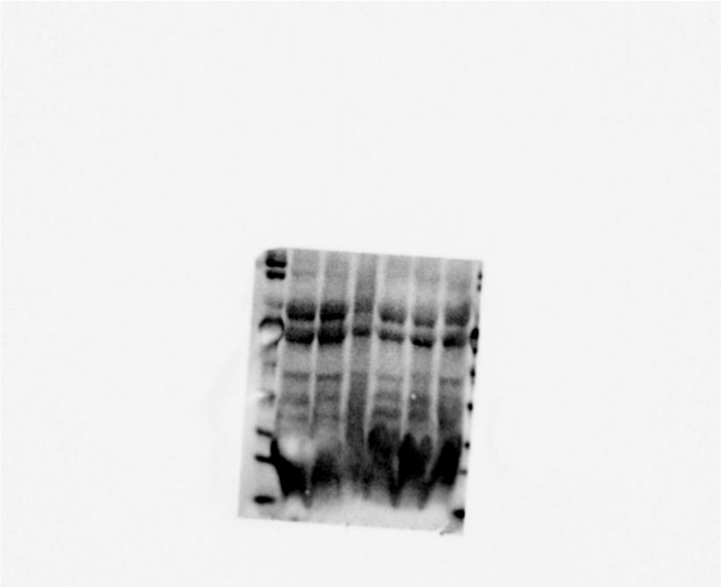

Pan-Kla

Figure 5

The sequence of protein loading was as follows:

N1, N2, N3, A1, A2, A3.

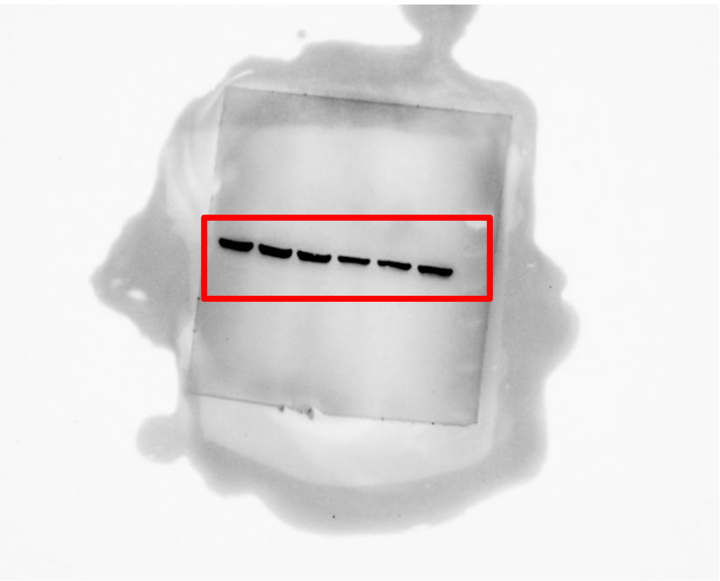

Pan-Kla (PGK2) 45kDa

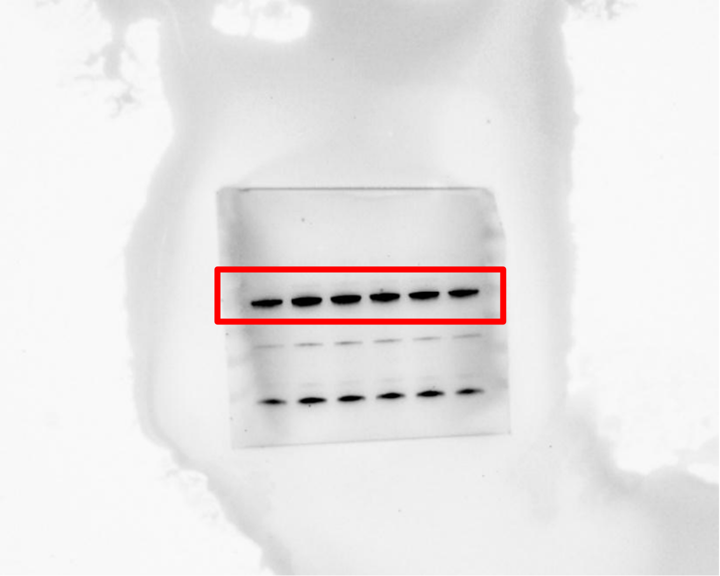

PGK2 45kDa

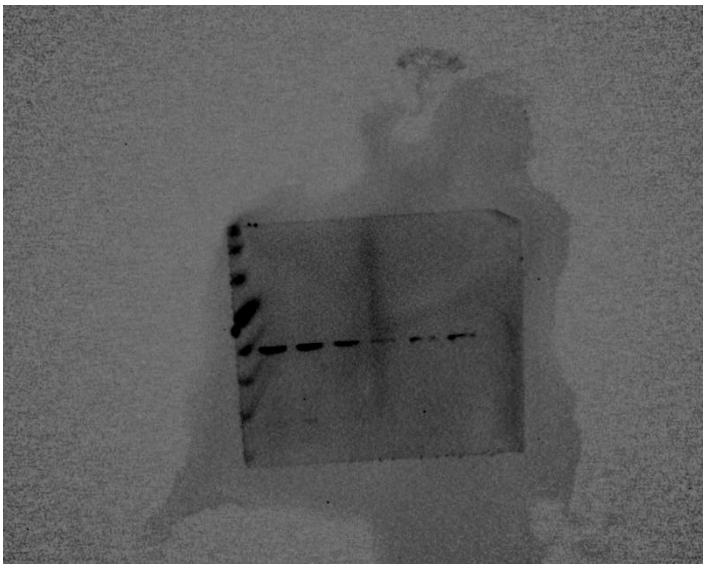

Pan-Kla (PGK2) 45kDa

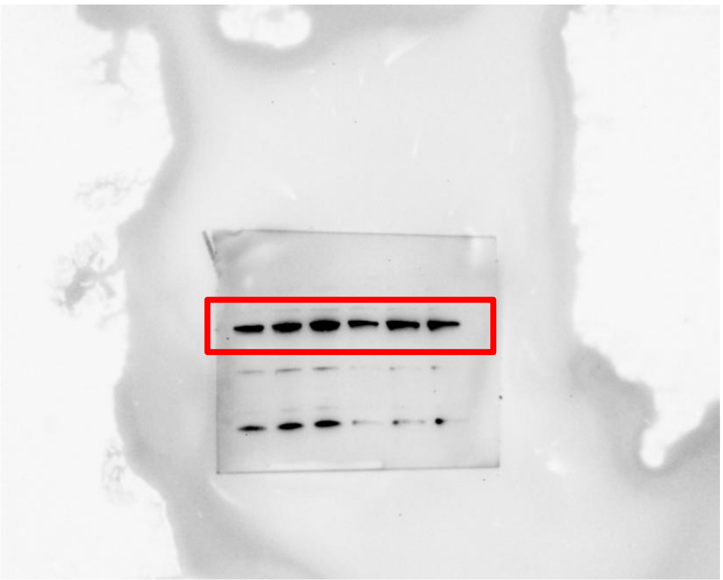

PGK2 45kDa

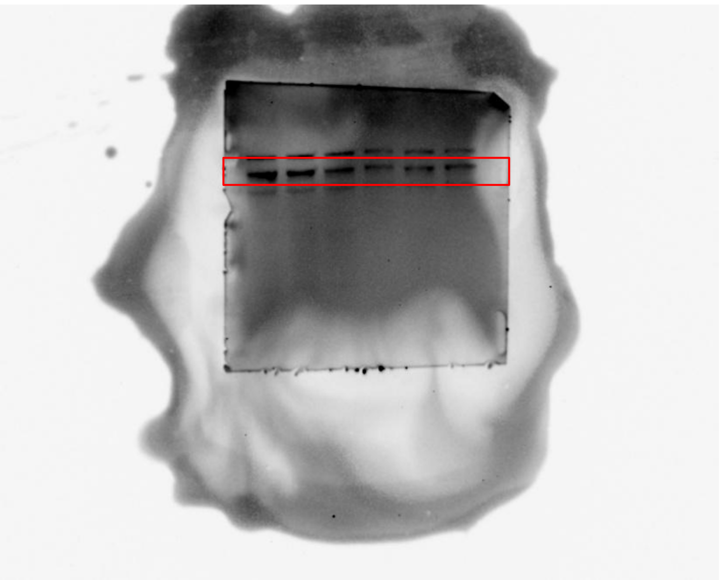

Pan-Kla (TEKT3) 55kDa

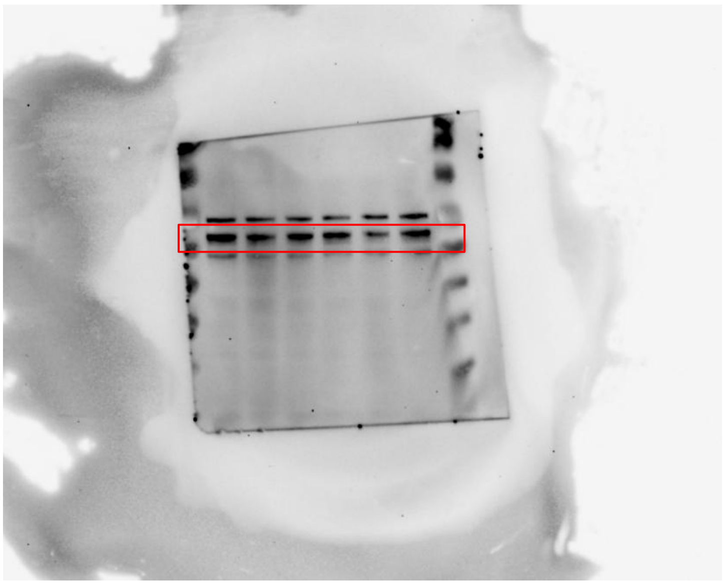

TEKT3 55kDa

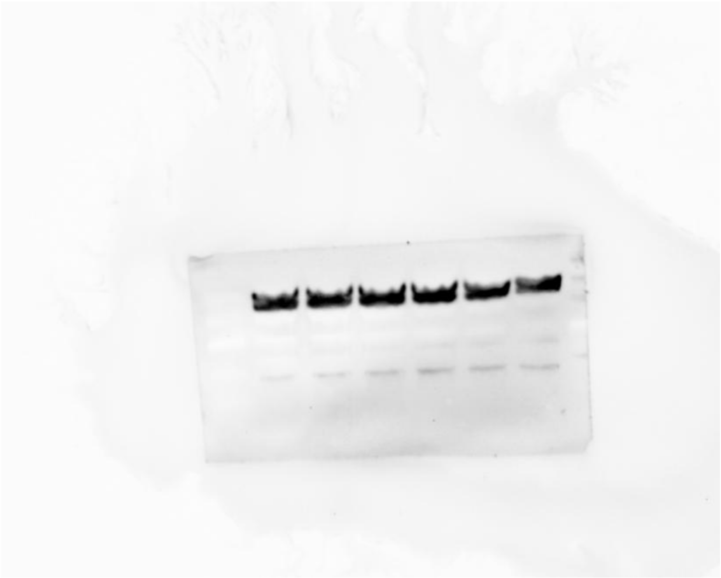

Pan-Kla (TEKT3) 55kDa

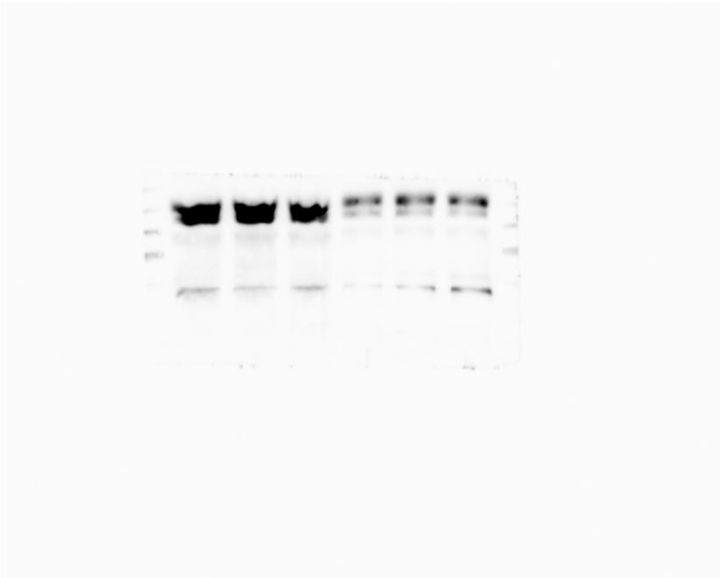

TEKT3 55kDa

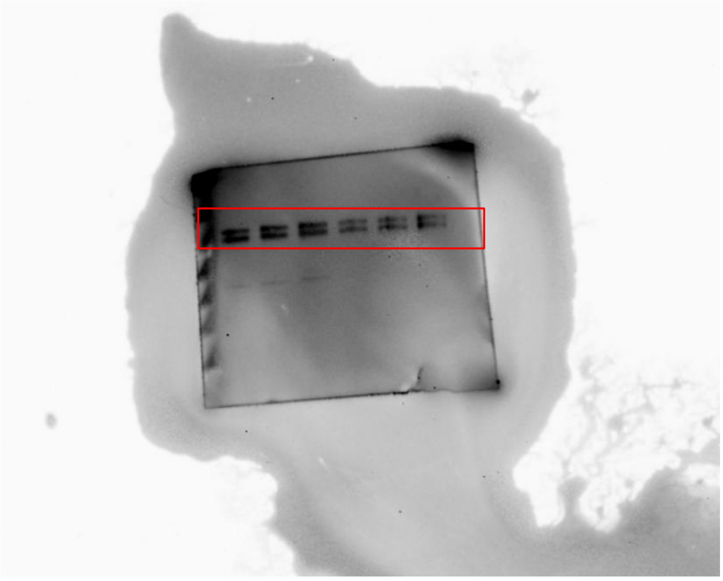

Pan-Kla (AKAP4) 82kDa

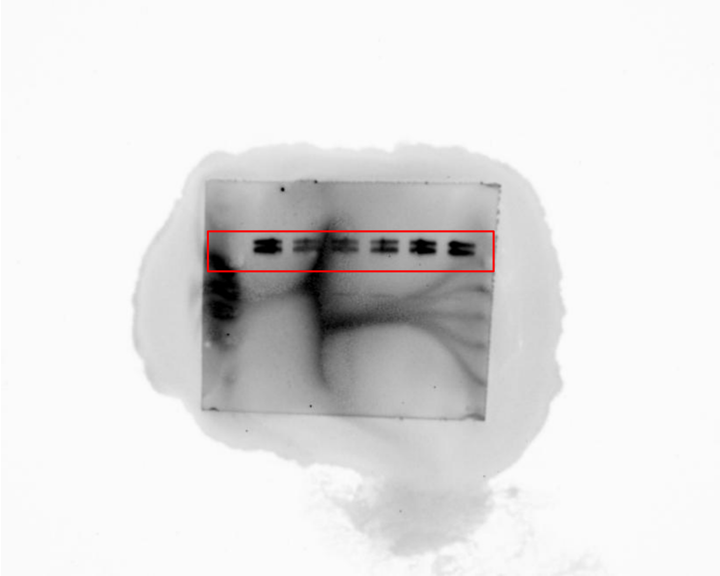

AKAP4 82kDa

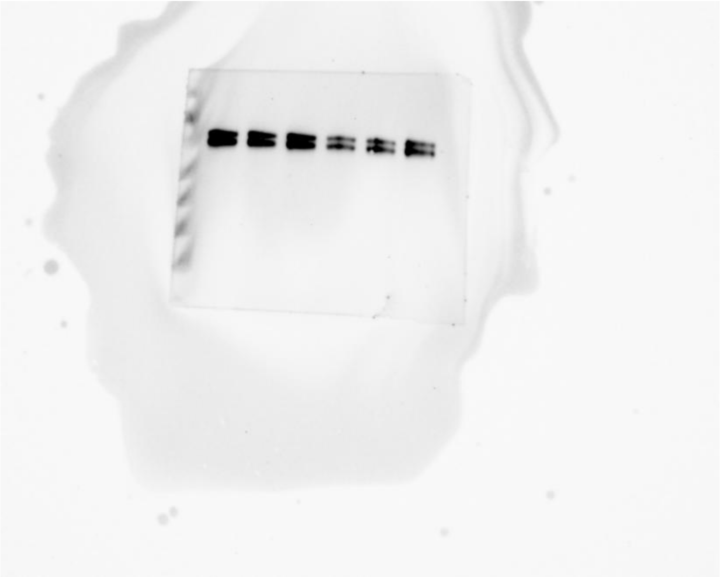

Pan-Kla (AKAP4) 82kDa

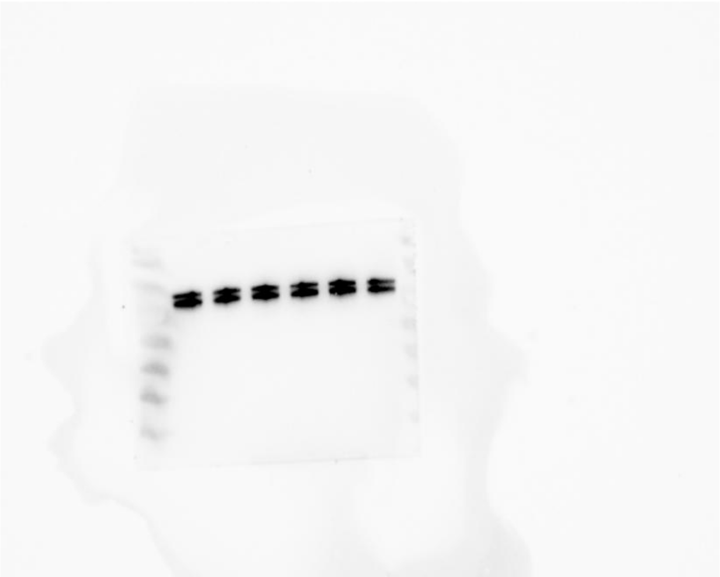

AKAP4 82kDa

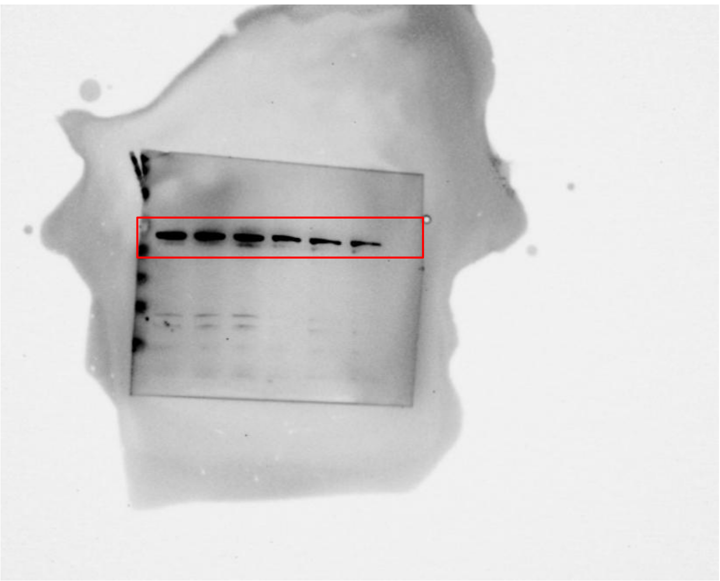

Pan-Kla (TUBA1A) 50kDa

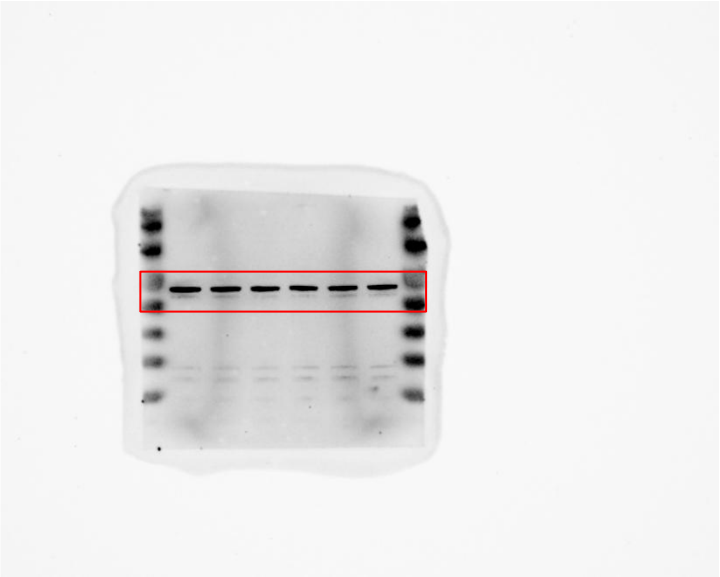

TUBA1A 50kDa

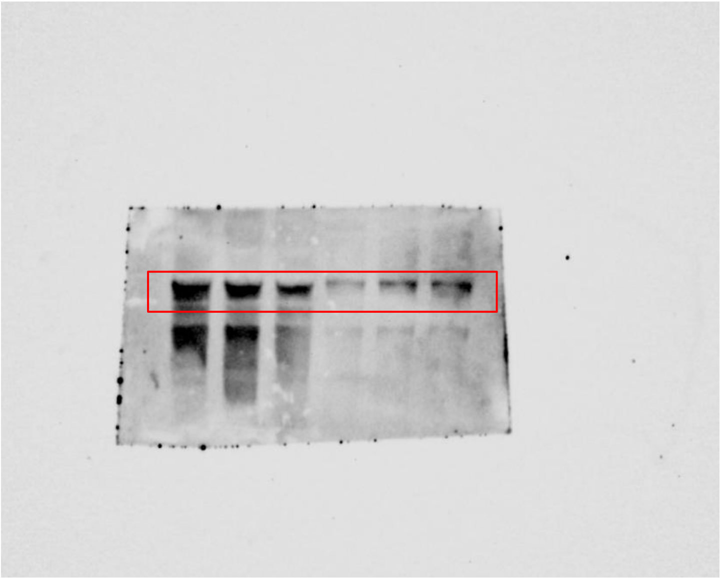

Pan-Kla (TUBA1A) 50kDa

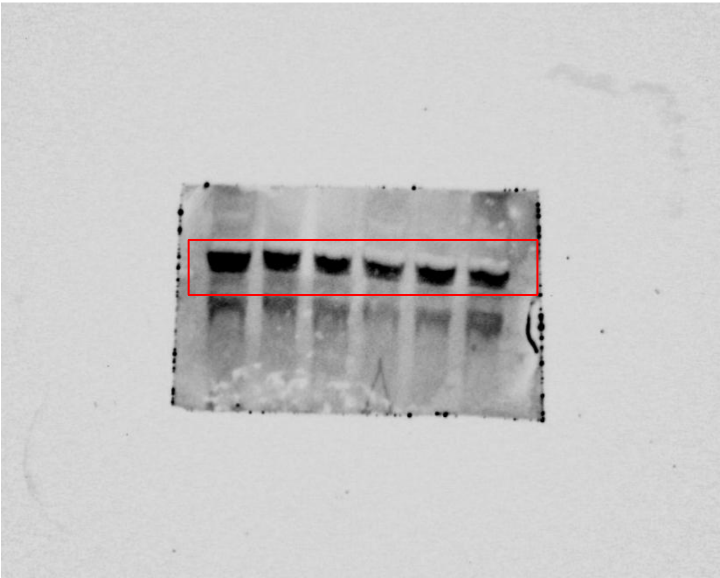

TUBA1A 50kDa

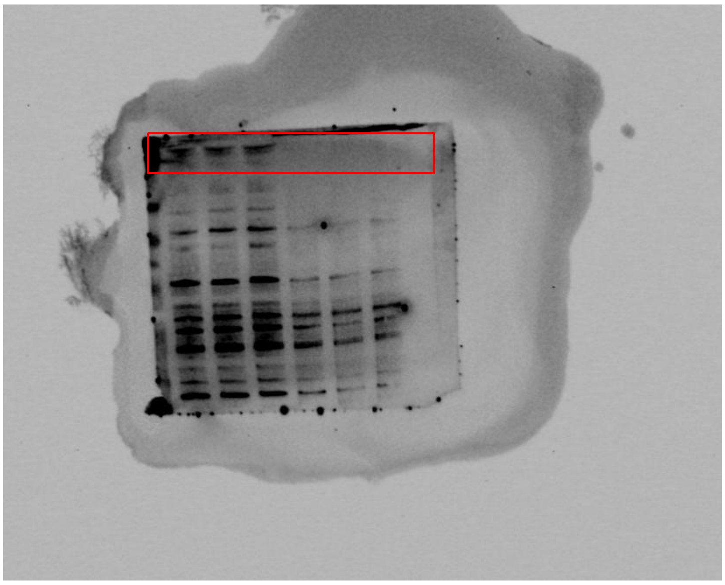

Pan-Kla (AKAP3) 100kDa

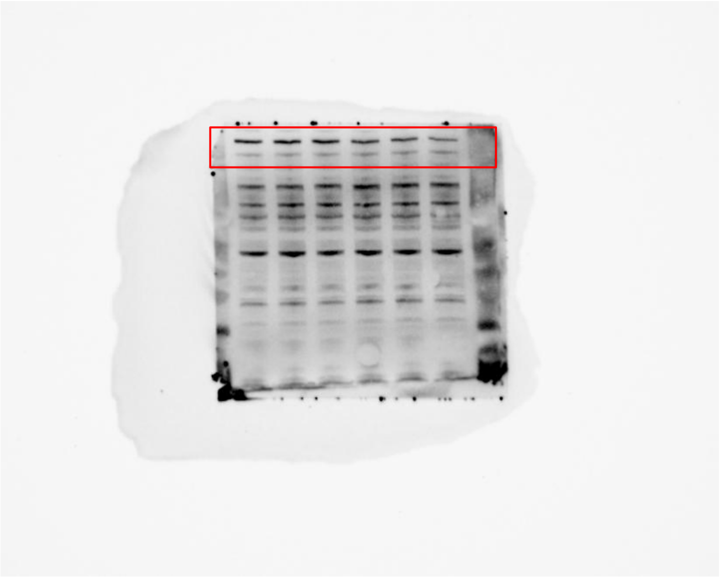

AKAP3 100kDa

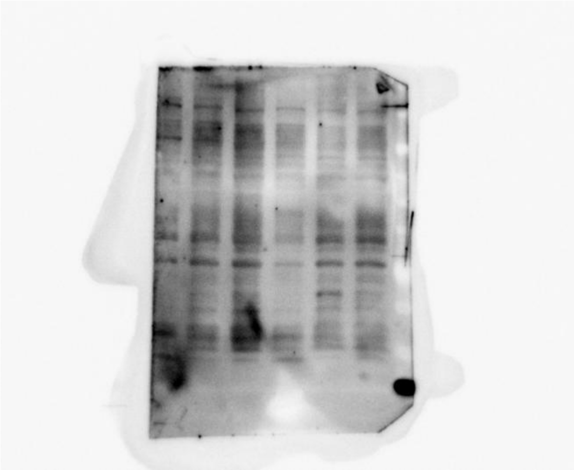

Pan-Kla (AKAP3) 100kDa

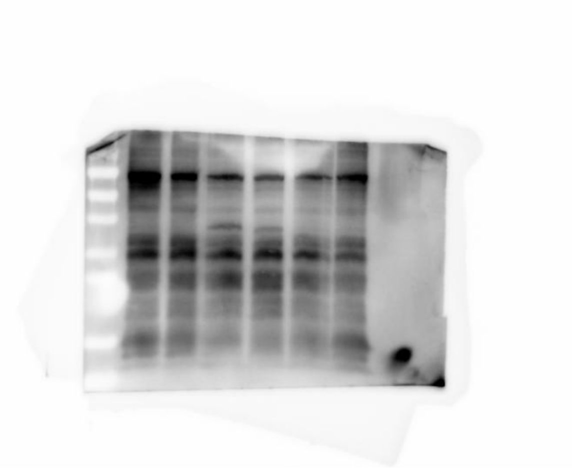

AKAP3 100kDa

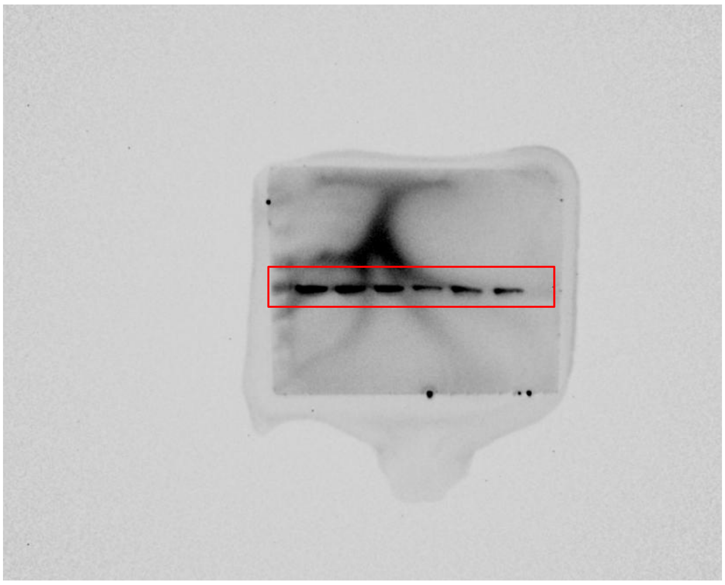

Pan-Kla (TUBB4B) 50kDa

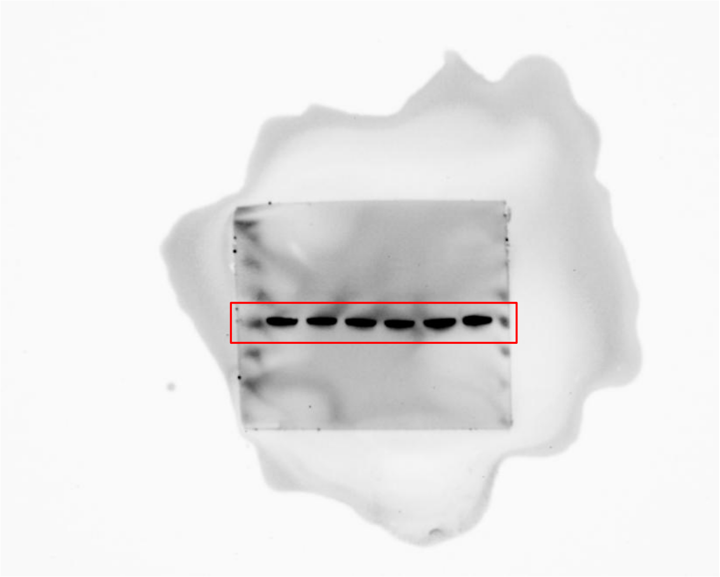

TUBB4B 50kDa

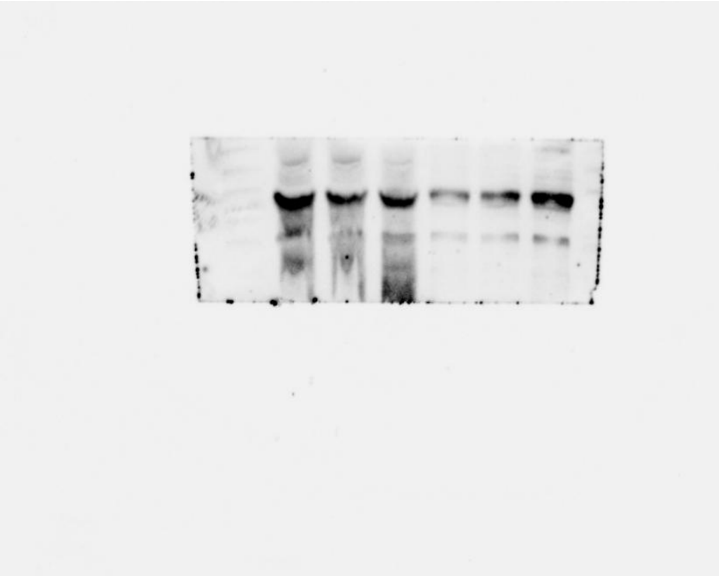

Pan-Kla (TUBB4B) 50kDa

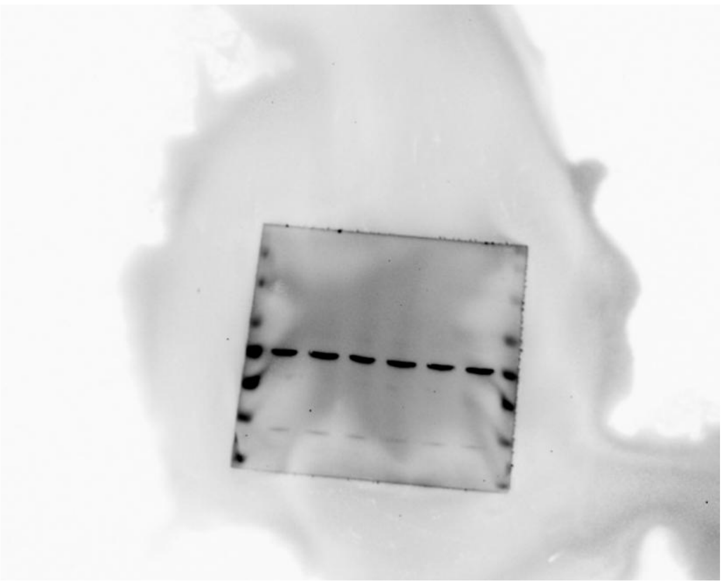

TUBB4B 50kDa
