# Supplementary figures and images for: Alterations in the protein lactylation landscape of sperm from patients with varicocele-associated asthenozoospermia
Source: Front Endocrinol (Lausanne). 2026 Jun 23;17:1791920. doi: 10.3389/fendo.2026.1791920 (PMC13337366; doi:10.3389/fendo.2026.1791920)

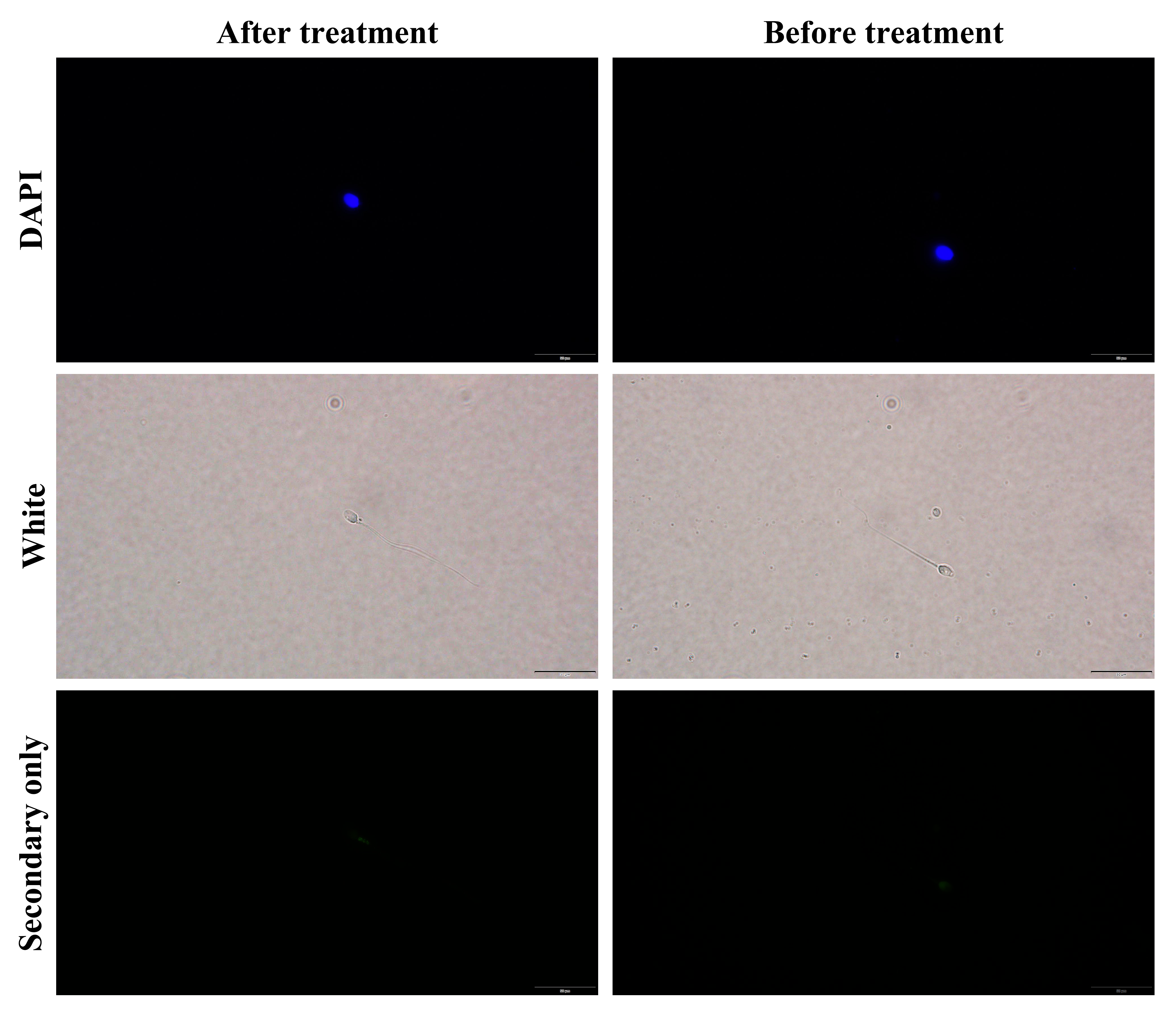

Supplement: Supplementary file 10 [file Image1.tif]

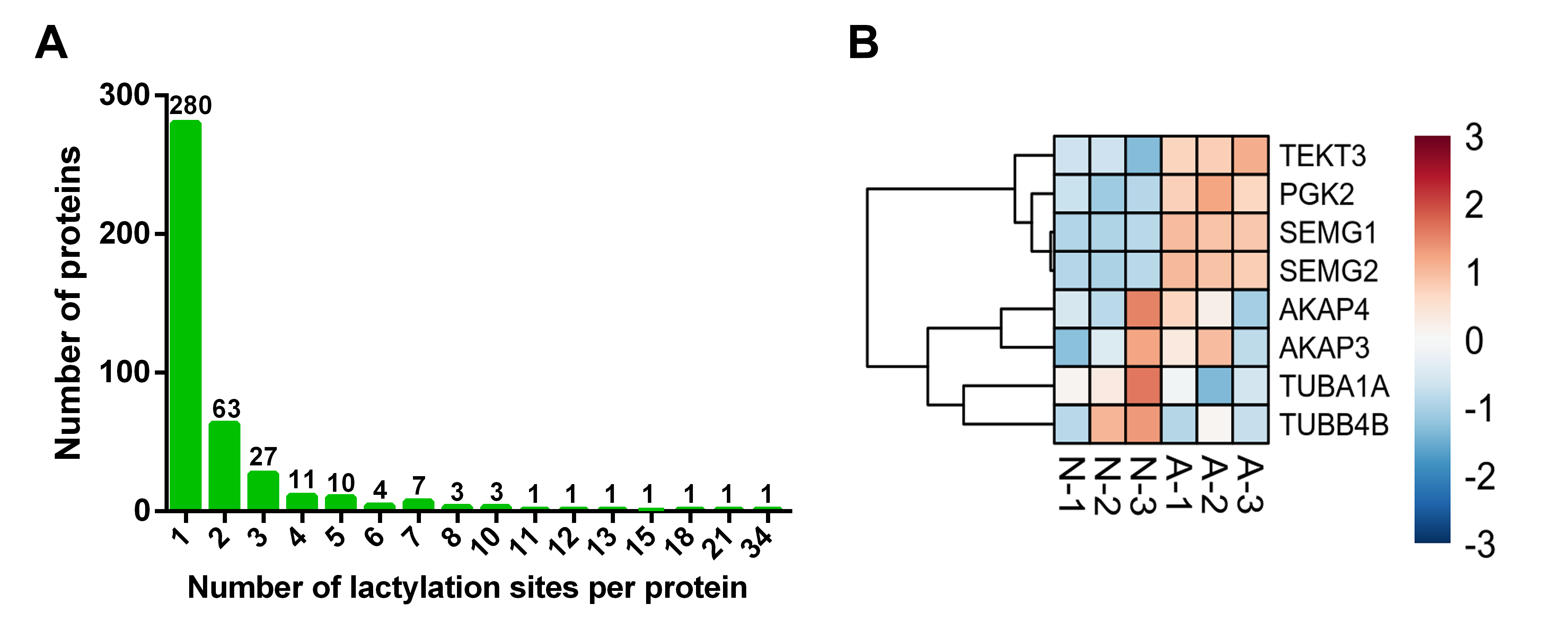

Supplement: Supplementary file 11 [file Image2.tif]
